# Biochemical–molecular–genetic biomarkers in the tear film, aqueous humor, and blood of primary open-angle glaucoma patients

**DOI:** 10.3389/fmed.2023.1157773

**Published:** 2023-05-26

**Authors:** Maria D. Pinazo-Durán, Vicente Zanón-Moreno, Carolina García–Villanueva, Alessio Martucci, Cristina Peris-Martínez, Jorge Vila-Arteaga, Jose J. García-Medina, Irene Andrés–Blasco, Alex Gallego–Martínez, Carlo Nucci, Julian García–Feijoo

**Affiliations:** ^1^Ophthalmic Research Unit “Santiago Grisolia”, Foundation for Research in Health and Biomedicine (FISABIO), Valencia, Spain; ^2^Cellular and Molecular Ophthalmobiology Group, Surgery Department, Faculty of Medicine and Odontology, University of Valencia, Valencia, Spain; ^3^Spanish Network of Inflammatory Diseases: REI-RICORS (RD21/0002/0032) of the Institute of Health Carlos III (ISCIII), Spanish Government, Madrid, Spain; ^4^Biosanitary Research Institute, Valencian International University (VIU), Valencia, Spain; ^5^Department of Ophthalmology, The University General Hospital, Valencia, Spain; ^6^Ophthalmology Unit, Department of Experimental Medicine, University of Rome “Tor Vergata”, Rome, Italy; ^7^Medical Ophthalmology FISABIO-FOM Center, Valencia, Spain; ^8^Department of Ophthalmology, University and Polytechnic Hospital “La Fe”, Valencia, Spain; ^9^Department of Ophthalmology, The General University Hospital “Morales Meseguer”, Murcia, Spain; ^10^Department of Ophthalmology and Optometry, University of Murcia, Murcia, Spain; ^11^Department of Ophthalmology, The University Clinic Hospital “San Carlos”, Madrid, Spain

**Keywords:** primary open-angle glaucoma, glaucoma neurodegeneration, biomarkers, molecules, genes, miRNAs

## Abstract

**Introduction:**

Glaucoma is a chronic neurodegenerative disease, which is the leading cause of irreversible blindness worldwide. As a response to high intraocular pressure, the clinical and molecular glaucoma biomarkers indicate the biological state of the visual system. Classical and uncovering novel biomarkers of glaucoma development and progression, follow-up, and monitoring the response to treatment are key objectives to improve vision outcomes. While the glaucoma imaging field has successfully validated biomarkers of disease progression, there is still a considerable need for developing new biomarkers of early glaucoma, that is, at the preclinical and initial glaucoma stages. Outstanding clinical trials and animal-model study designs, innovative technology, and analytical approaches in bioinformatics are essential tools to successfully uncover novel glaucoma biomarkers with a high potential for translation into clinical practice.

**Methods:**

To better understand the clinical and biochemical-molecular-genetic glaucoma pathogenesis, we conducted an analytical, observational, and case-comparative/control study in 358 primary open-angle glaucoma (POAG) patients and 226 comparative-control individuals (CG) to collect tears, aqueous humor, and blood samples to be processed for identifying POAG biomarkers by exploring several biological pathways, such as inflammation, neurotransmitter/neurotrophin alteration, oxidative stress, gene expression, miRNAs fingerprint and its biological targets, and vascular endothelial dysfunction, Statistics were done by using the IBM SPSS 25.0 program. Differences were considered statistically significant when *p* ≤ 0.05.

**Results:**

Mean age of the POAG patients was 70.03 ± 9.23 years, and 70.62 ± 7.89 years in the CG. Malondialdehyde (MDA), nitric oxide (NO), interleuquin (IL)-6, endothelin-1 (ET-1), and 5 hydroxyindolacetic acid (5-HIAA), displayed significantly higher levels in the POAG patients vs. the CG (*p* < 0.001). Total antioxidant capacity (TAC), brain derived neurotrophic factor (BDNF), 5-hydroxy tryptamine (5-HT), solute carrier family 23-nucleobase transporters-member 2 (*SLC23A2*) gene, and the glutathione peroxidase 4 (*GPX4*) gene, showed significantly lower levelsin the POAG patients than in the CG (*p* < 0.001). The miRNAs that differentially expressed in tear samples of the POAG patients respect to the CG were the hsa miR-26b-5p (involved in cell proliferation and apoptosis), hsa miR-152-3p (regulator of cell proliferation, and extracellular matrix expression), hsa miR-30e-5p (regulator of autophagy and apoptosis), and hsa miR-151a-3p (regulator of myoblast proliferation).

**Discussion:**

We are incredibly enthusiastic gathering as much information as possible on POAG biomarkers to learn how the above information can be used to better steer the diagnosis and therapy of glaucoma to prevent blindness in the predictable future. In fact, we may suggest that the design and development of blended biomarkers is a more appropriate solution in ophthalmological practice for early diagnosis and to predict therapeutic response in the POAG patients.

## 1. Introduction

Glaucoma is a kind of progressive eye disease that is precisely characterized by optic nerve degeneration (OND), which manifests itself in the ocular fundus as optic disc cupping due to retinal ganglion cell (RGC) loss, and the corresponding visual field (VF) defects, being these the structural and functional reference landmarks of the disease (1–3). Glaucoma is a neurodegenerative disease that affects millions, and is the first global cause of irreversible blindness. In Europe, primary open-angle glaucoma (POAG) increased odds correlated to aging. Moreover, POAG prevalence is expected to grow in Europe because strength rises in the older population (4, 5). POAG is the most prevalent glaucoma form, characterized by intraocular pressure (IOP) elevation (the most relevant risk factor for the disease), with a typical anterior chamber angle appearance, and no other ocular identifiable comorbidity that may be causing ocular hypertension (OHT) (6, 7). Aging African–Caribbean and Hispanic races, myopia, thinner cornea, familial glaucoma history, and other conditions are important risk factors for the development and progression of POAG, which have to be kept in mind when gathering information for a complete clinical history (6, 8–10). Moreover, in a recent Spanish–Portuguese population study, overweight/obesity, migraine, asthma, and smoking have been shown to be significant risk factors for conversion from OHT to POAG (11). Imaging techniques for glaucoma, such as anterior and posterior optical coherence tomography (OCT) and OCT angiography (OCTA), OCT elastography, the oximetry, and hyperspectral image, fluorescence lifetime imaging ophthalmoscopy, and detection of apoptotic RGCs (12, 13), have been arising in the past years. Also, the VF fully automated innovating techniques (1–3, 5), as the combination of perimetry with the application for the colorimetric analysis of optic nerve head (ONH) images (which topographically assesses the cup and the presence of hemoglobin), the Laguna-ONhE, improved using five deep learning models (14), have been recently described.

The IOP reduction diminished the risk of progression in the glaucoma patients involved in The Ocular Hypertension Treatment Study (OHTS) (15, 16) and is currently the only affordable glaucoma therapy. First-line treatment is constituted by the hypotensive eye drops of prostaglandin analogs and β-blockers (1, 10, 17). However, the glaucoma progression rate and the risk of visual impairment are arduous to foresee individually because a vital part of the glaucoma patients appropriately receiving hypotensive medical–laser–surgical treatment still undergo visual impairment and blindness, while others remain stable even with having higher IOP (18–20). Because of this, up-today, glaucoma has no cure.

Experimental models of glaucoma have been extensively used (21–26). Pre-clinical glaucoma models have been mainly carried out in rodents, which have successfully reproduced the human glaucoma milestones: specific RCGs and axons damage and death, anterior and posterior eye segment changes, and elevated IOP (22, 23, 25, 26). Therefore, in animal glaucoma models, it has been demonstrated that raised chronic IOP always precedes glaucomatous OND. Many animal models of OHT have mimicked POAG by developing moderated and sustained IOP increases that induced apparent retinal and optic nerve damage (22, 23, 25, 26).

Elevated IOP induces cellular stress with the result of mitochondrial failure, autophagy, and apoptosis (27–30). Aging is a pivotal risk factor for oxidative stress (OS) and dysregulation of inflammation (INF) (13, 25, 27–31). Moreover, morphologic and functional changes in the trabecular meshwork are mediated by OS and INF (13, 32). It has also been suggested that systemic endothelial and autonomic dysfunction are present in POAG (33) as well as reduced blood flow and vascular dysfunction (VD) have been recognized as glaucoma features (34). In addition, the role of neurotransmitters such as glutamate, glycine, dopamine, serotonin, etc., and neurotrophins, such as the brain-derived neurotrophic factor (BDNF), neurotrophin-3 (NT3), and neurotrophin-4/5 (NT4/5) in glaucoma, has also been extensively investigated (35, 36). The POAG gene candidates have also been described. Myocilin (MYOC), optineurin (OPTN), WD repeat domain 36, (WDR36), cytochrome P450, family 1, subfamily B, polypeptide 1 (CYP1B1), glutathione S-transferase mu 1 (GSTM1), and Neurotrophin (NTF4) have been widely linked to POAG (37, 38). The small single-stranded microRNAs (miRNAs) can regulate the expression of mRNAs/proteins within a cell and have been extensively implicated in numerous diseases, including the most prevalent ocular disorders (39). Some specific miRNAs regulate IOP (40), and others have been proposed for the diagnosis of glaucoma (41). Despite the aforementioned facts, the molecular and cellular hallmarks underlying OND in glaucoma still remain unsolved.

There is a relevant need for finding/validating imaging, biochemical, molecular, and genetic glaucoma biomarkers that help improve the clinical diagnosis for early detection, as well as for monitoring glaucoma therapy and disease progression.

In this study, we described some validated classical biomarkers for POAG diagnosis and treatment and potential new biomarkers with higher sensitivity based on clinical and experimental glaucoma research.

## 2. Materials and methods

### 2.1. Focused question

To better understand the clinical and molecular–genetic glaucoma pathogenesis, we conducted a collaborative multicenter, analytical, observational, case–comparative/control study of 625 participants, classified according to the inclusion/exclusion criteria, that were initially recruited from the glaucoma sections at the ophthalmological departments of the following hospitals: General of Valencia; “Dr. Peset” of Valencia; University of Rome “Tor Vergata” Rome, Italy; University and Polytechnic “La Fe” of Valencia; “Morales Meseguer” of Murcia; and Clinic “San Carlos” of Madrid. The study volunteers agreed to participate in the study and signed the informed consent form. In this study, we adhered to the principles of the Declaration of Helsinki is universally known in science and the Ethics Committee standards of the study centers (2020–2022). All clinical research requirements to maintain the data privacy of our study participants were explicitly met.

### 2.2. Eligibility criteria

Ophthalmologists from the glaucoma sections performed a systematized ocular examination of the potential participants of both sexes, aged 40–80 years, who had an appointment for the eye clinic. A total of 625 individuals were selected by a non-random consecutive sampling procedure. Most suitable study participants were chosen after ensuring their health status and ocular condition in agreement with the inclusion and exclusion criteria, as shown in Table 1. The suitable volunteers were classified into two groups: (1) patients with POAG diagnosis (POAGG; *n* = 380) and (2) individuals without glaucoma, as a comparative–control group (CG; *n* = 245). The whole sample of POAG patients was under glaucoma treatment (hypotensive eye drops, laser, or glaucoma surgery), depending on the glaucoma stage and personal characteristics. The final sample size of our study participants was (358 POAG patients and 226 CG individuals). The reduction from the initial number of the recruited sample was due to volunteer dropout, clinical findings/unique or unusual complications that recommended excluding the participant, and/or sampling transport or laboratory processing.

**Table 1:** Inclusion and exclusion criteria for the study participants.

**Inclusion**	**Exclusion**
Individuals aged 40–80 years	Individuals under 40 years of age
Accurate diagnosis of POAG for the corresponding group	Other glaucoma type
Non-glaucomatous healthy individuals for the comparative–control group of participants (CG)	Patients experiencing other ophthalmological diseases and/or comorbidities. Patients receiving local or systemic treatment that may interfere with the study. Eye laser surgery in the previous 12 months.
Precise and complete data on the medical history. Psycho-physical status that permits for participation in the study	History, including previous diagnoses that do not fit with the study purpose Unfeasibility of having a thorough and complete clinical history and being unable to participate.

POAG, primary open-angle glaucoma; CG, comparative controls.

### 2.3. Proceedings of the clinical approaches

Each participant was interviewed to determine the social and demographic factors, systemic comorbidities, and glaucoma medications. Data corresponding to this part were recorded as DEMO in a Microsoft Excel spreadsheet that was explicitly reviewed by the study coordinators.

Ophthalmological examination was performed by combining the IOP measurements (using Goldmann applanation tonometry Haag–Streit AT 900; Haag–Streit Köniz, Switzerland), morphological measurements [indirect gonioscopy through a slit lamp (IMAGEnet, Topcon, Barcelona, Spain) with the Goldmann 3-mirror lens, to demonstrate an open anterior chamber angle; ocular fundus exploration through a slit-lamp with a 78 D lens; and optical coherence tomography (OCT) examination (Cirrus spectral–domain OCT, Carl Zeiss Meditec, Inc., Madrid, Spain)], as well as the functional VF performance, using the 24-2 Swedish interactive threshold algorithm, Humphrey field analyzer (Carl Zeiss Meditec, Inc., Madrid, Spain). In this context, best-corrected visual acuity (BCVA) obtained by the logarithm of the minimum angle of resolution (LogMAR) for each eye and the IOP determination (three consecutive times) were registered during the ophthalmological visit. It has to say that only the ophthalmologists involved in the present study were responsible for the measurements. Normal IOP is considered as the IOP two standard deviations (SDs) above the normal, i.e., 21 mm Hg, and the IOP above this level was defined as OHT. The IOP values were registered in millimeters of mercury as the mean ± SD for three determinations for each participant's eye. To determine the central corneal thickness (CCT), the hand-held ultrasonic pachymetry (Reichert^®^ iPac^®^ Reichert/Ametek, Munich, Germany) was used, and three independent measurements were performed in a random sequence of 3 min from each other. Normal CCT was estimated at 533 μm and registered as the mean ± SD of three determinations for each participant's eye. For the CG, the IOP had to be lower than 21 mmHg, with normal visual fields, optic disc, and retinal nerve fiber layer (RNFL) in absence of another ocular or systemic disease. Moreover, glaucomatous OND was considered when including specific ONH alterations, such as neuroretinal rim thinning, splinter hemorrhages, peripapillary nerve fiber loss, asymmetry of cupping between patient eyes, and parapapillary atrophy, among others. We recorded the data into a Microsoft Excel spreadsheet as “OPHTHAL,” which was reviewed by two independent ophthalmologists.

Three types of biosamples were collected from the study participants for the programmed experiments, tears, aqueous humor, and blood. (1) Reflex tears were collected (30 μl) from the two study groups by a gentle rubbing of the inferior meniscus and external canthus of each eye without instilling anesthetics, as previously described (42–44), by using a Microhematocrit capillary tube, appropriately labeled, which was immediately transferred into micro-Eppendorf tubes and stored at −80°C until processing. (2) Aqueous humor was collected (100 μl) at the time of glaucoma surgery, under an operating microscope by means of a Rycroft cannula, through the incision for performing classic trabeculectomy, valvular procedures, or microincisional glaucoma surgery (MIGS), with special care to avoid contamination. Samples were immediately frozen at −85°C until processing, as described elsewhere (45, 46). Also, at the time of surgery, the aqueous humor was collected from individuals programmed for non-complicated cataract surgery with the same described protocol (27–29, 40–42) that were considered the comparative–control participants. (3) Peripheral blood samples were collected, from the two study groups, under fasting conditions at 8:00 a.m. from the antecubital vein into 4.5 ml ethylene-diamine-tetra-acetic acid (EDTA) or sodium citrate vacutainer tubes (Becton Dickinson, Auckland, New Zealand), as an anticoagulant. The EDTA tube (purple cap) was used to analyze gene expression. The sodium citrate tube (purple cap) was centrifuged at 3,000 rpm/10 min to obtain the plasma fraction, which was aliquoted and stored at −80°C until processing to diverse analyses, as previously shown (47, 48). All experiments were performed in duplicate at the laboratories of the Ophthalmic Research Unit “Santiago Grisolía” and the Department of Surgery of the University of Valencia (Valencia, Spain).

The biochemical, molecular, and genetic actors that were assayed in this study were malondialdehyde (MDA), total antioxidant capacity (TAC), nitric oxide (ON), interleukin-6 (IL-6), endothelin 1 (ET1), brain-derived growth factor (BDNF), serotonin or 5-hydroxy tryptamine (5-HT), and its metabolite the 5 hydroxyindolacetic acid (5-HIAA), specific genes related to antioxidant status (SLC23A2 gene and GPX4 gene), and specific miRNAs.

The proceedings are described below.

- *Determination of lipid peroxidation by-products*. This was measured by the MDA/TBARs Assay Kit (Reference: KB-03-016, BioQuochem, Asturias, Spain), based on the measurement of thiobarbituric acid reactive substances (TBARS) formed after the reaction of MDA with thiobarbituric Acid (TBA). The assay was done under high-temperature conditions (90°C for 60 min), and the product of the reaction was measured in a colorimetric way using a 532 nm absorbance value for the plasma samples. The concentration was calculated by extrapolating all data in the standard curve as reported (27–31, 45–47).
- *Determination of TAC*. This was measured by the antioxidant assay kit (Reference: 709001, Cayman Chemical Company, Ann Arbor, MI, USA) based on the antioxidant capacity to inhibit the 2, 2′-azino-di-[3-ethylbenzthiazoline sulphonate] oxidation to 2, 2′-azino-di-[3-ethylbenzthiazoline sulphonate] radical solution by the metmyoglobin, as published (27–31, 46– 49). The reaction's product was measured colorimetrically using a 750-nm absorbance value for the plasma samples. The concentration was calculated by extrapolating all data in the standard curve.
- *Determination of NO*. The total nitric acid was determined using a commercial preparation by R&D Systems. This essay was based on the enzymatic conversion of nitrate to nitrite by means of nitrate reductase enzyme. After the reaction, the colorimetric determination of the nitrite is carried out by Griess' reaction, which is based on a two-stage diazotization reaction: (1) acidification of NO_2_ to produce a nitrosating agent and (2) reaction of this agent with sulfanylic acid to produce a diazonium ion which will be joined to N-(1-naphthyl) ethylenediamine to form a chromophore which absorbs light at 540–570 nm, and which is measurable in the aqueous humor samples (50).
- *Determination of 5-HT and 5-HIAA*. Samples were diluted 1:3 v/v in 0.2N perchloric acid, filtered through a 0.2 μm Nylon microfilter (Costar, Cambridge, MA) by centrifugation (10,000 rpm for 5 min at 4°C), and analyzed by high-performance liquid chromatography (HPLC) with electrochemical detection (ECD), according to a modified method of Audhya, Adams, and Johansen (51). Analyses were performed using a Gilson Medical Electronics HPLC system (Middleton, WI) with an LC-234 auto-injector equipped with an LC3-07 delivery pump and with an LC-142 electrochemical detector under reversed phase conditions with a Supelcosil LC 7.5 cm × 4.6 cm, 3-mm column (Supelco; Sigma-Aldrich, Bellefonte, PA). The software used was a 712 HPLC system controller data version 1.30 management (Gilson Medical Electronics). Compounds were eluted isocratically over an 18-min runtime at a 1 ml/min flow rate. The mobile phase consisted of 70 mM potassium dihydrogen phosphate buffer (pH adjusted to 3.0 with phosphoric acid), 1 mM 1-hepatosulfonic acid, 107.5 mM sodium EDTA, and 10% methanol. Sample injection was 20 ml, and the electrochemical detector was recorded with a glassy carbon working electrode set at +0.75 V. Identification was performed by comparison with standard retention times determined by injections of standard mixture run at given intervals between sample analyses. Quantification was made using the calibration curve standards with 5-HT (*r* = 0.0004) and 5-HIAA (*r* = 0.0003). Samples were injected in duplicate, and the amount of each compound was expressed as mean ± SE in ng/ml of the corresponding blood or aqueous humor sample.
- *Determination of IL-6*. Tears, aqueous humor, and plasma samples were used to determine the cytokine expression in POAG patients vs. the CG. This was performed using the Human IL-6 ELISA Kit (Ref: EH2IL6, Invitrogen, Vienna, Austria), which was based on an *in vitro* enzyme-linked immunosorbent assay (ELISA) for the quantitative measurement of human IL-6.An IL-6 molecule is binding for two antibodies: (1) monoclonal antibody specific for IL-6 and (2) acetylcholinesterase:Fab' conjugate. The enzymatic activity of acetylcholinesterase is measured at 450 nm. The concentration of the IL-6 is proportional to the amount of bound conjugate as widely reported (42–44, 52, 53).
- *Determination of ET1*. We used a commercial kit for human endothelin-1 (BI-20052, Biomedica Gruppe, Vienna, Austria). Using this kit, by the action of the Endothelin Converting Enzyme-1 (ECE-1), the pro-ET molecule (or big ET) is split into two fractions: the active ET (21 amino acids) and an inactive C-terminal fragment. Using a double-antibody sandwich technique, the active ET binds the two antibodies. A conjugate is formed, producing a yellow color as an indicator, which absorbs at 450 nm. The intensity of this color is proportional to the amount of bound conjugate, which in turn is proportional to the ET concentration in the aqueous humor samples as described (54).
- *Determination of BDNF*. We used the Human BDNF Immunoassay Kit (DBD00, R&D Systems Inc., MN, USA). The kit is based on a double-antibody sandwich technique. The BDNF present in samples is bound to the two antibodies, forming a conjugate. This conjugate reacts with a substrate solution, and the color is developed proportionally to the BDNF concentration. The intensity of the color is measured by spectrophotometry at 540 nm for the aqueous humor samples, as described (55).
- *Gene expression assays*. Whole blood samples were obtained from each participant and collected into EDTA tubes. Total RNA was isolated from blood samples by the Trizol method. Then, 300 ng of total RNA (integrity number: RIN > 7) were converted into cDNA by reverse transcription using the High-Capacity RNA-to-cDNA™ Kit (Applied Biosystems, Foster City, CA, USA). The relative SLC23A2 and GPX4 gene expression was analyzed by real-time PCR using a 7900HT Sequence Detection System (SDS; Applied Biosystems^®^, Madrid, Spain). TaqMan gene expression assays were used for both target (SLC23A2, GPX4) and internal control (18S rRNA) genes (Applied Biosystems^®^, Spain). Samples were assayed in duplicate. The expression values were calculated by the double delta Ct formula as previously reported (56, 57). The results were expressed as fold changes in gene expression for each group and subgroup at baseline.
- *miRNA expression assays*. On the day of processing, samples were defrosted and prepared for RNA extraction using the miRNeasy Mini Kit (QIAGEN Inc., Hilden, Germany). Briefly, purification is based on spin column chromatography using a proprietary resin as the separation matrix. Small RNAs are separated from other cellular components, such as proteins without using phenol or chloroform. The quality and quantity of total RNA obtained from tears were assessed using a Bioanalyzer 2100 (Agilent^®^ Technologies, Inc., Santa Clara, CA, USA) and the RNA 6000 Nano Kit (Agilent^®^ Technologies, Inc.). RNA libraries were prepared using NEBNext^®^ Multiplex Small RNA Library Prep Set for Illumina^®^ (#E7300 y #7580; New England BioLabs^®^, Inc., Ipswich, MA, USA), according to the manufacturer's protocol (https://international.neb.com/protocols/2018/03/27/protocol-for-use-with-nebnext-small-rna-library-prep-set-for-illumina-e7300-e7580-e7560-e7330). According to the guidelines for low RNA concentration samples, the adapters and RT primers were diluted 1:2 with nuclease-free water, and 15 cycles were used for the amplification by PCR. The indexed libraries were purified using the QIAquick^®^ PCR Purification Kit (#28104, QIAGEN^®^, Hilden, Germany). Library quality control was assessed using a 4200 TapeStation (Agilent^®^ Technologies, Inc.) and High Sensitivity D1000 Kit (Agilent^®^ Technologies, Inc.). The miRNA fraction of each library (120–200 bp) was collected using the Pippin Prep System (Sage Science, Inc., Beverly, MA, USA) following the manufacturer's guidelines and using 3% agarose dye free gel cassettes with internal standards (Marker P) (Sage Science #CDP3010). miRNAs were quantified using a 4200 TapeStation (Agilent^®^ Technologies, Inc.) and High Sensitivity D1000 Kit (Agilent^®^ Technologies, Inc.) prior to normalization and pooling. Sequencing was performed on a NextSeq 500 System (Illumina, Inc., San Diego, CA, USA) with a Mid-Output flow cell for 150-cycle reads, obtaining about 3.5 million reads per sample. FASTQ file quality was assessed using the FASTQC tool (https://www.bioinformatics.babraham.ac.uk/projects/fastqc/). Adapters and low liability reads were removed. At this point, non-coding RNAs previously described in the ENSEMBL database were selected and characterized. Statistical analyses were performed using Limma and edgeR packages deposited in Bioconductor (www.bioconductor.org). A predictive analysis based on receiver operating characteristic (ROC) curves was performed to select those miRNAs showing an area under the curve (AUC) >0.75. Subsequently, an analysis of the main components (PCA) was performed. The proceeding for searching the target genes of the selected miRNAs was reported elsewhere (58–60).

### 2.5. Statistical processing

All data were statistically processed using the IBM SPSS 25.0 program. The normal distribution of the quantitative variables was

verified using the Shapiro-Wilk test (subgroups) and Kolmogorov-Smirnov test (main groups). Qualitative variables were described by absolute and relative frequencies. Quantitative variables were defined using the mean and standard deviation (normal distribution) or median and interquartile range (non-normal distribution). Differences between quantitative variables were analyzed using the Student's *t*-test for independent samples and ANOVA (normal variable) or the Mann–Whitney and Kruskal–Wallis U test (non-normal variable). The association between qualitative variables was determined using the Chi-square test or Fisher's exact test. The correlation between two quantitative variables was analyzed using the Pearson correlation coefficient (normal distribution) or the Spearman correlation coefficient (non-normal distribution). The differences were considered statistically significant when *p* ≤ 0.05.

## 3. Results

Absolute number of study participants was 584, which was subdivided into POAG patients (*n* = 358) and CG individuals (*n* = 226). The mean age was 70.03 ± 9.23 years in the POAGG and 70.62 ± 7.89 years in the CG. Gender distribution was 70% women in the POAG and 52% in the CG.

All POAG patients showed IOP elevation, increased optic disc excavation, ONH damage, and/or altered visual fields, and these participants were all under glaucoma treatment. The ophthalmological parameters of the study participants are shown in Table 2. Moreover, individuals conformed to the control–comparative group of non-glaucoma eyes and did not show any of the above glaucoma hallmarks.

**Table 2:** Ophthalmological characteristics of the study participants.

**Clinical variables**	**POAGG**	**CG**	***p*-value**
IOP (mm Hg)	19 ± 2	15 ± 2	< 0.0001
CCT (mm)	534 ± 31	579 ± 38	< 0.0001
Average C/D ratio	0.6 ± 0.02	0.04 ± 0.04	< 0.0001
Vertical C/D ratio	0.6 ± 0.1	0.1 ± 0.01	< 0.0001
Average RNFL thickness (μm)	73 ± 12	91 ± 13	< 0.0001
Rim area (μm^2^)	5 ± 0.9	1 ± 0.6	< 0.0001
RGCs density	66 ± 10	92 ± 9	< 0.0001

POAGG, primary open-angle glaucoma group; CG: comparative–control group; IOP, intraocular pressure; CCT, central corneal thickness; C/D, cup/disk; RNFL, retinal nerve fiber layer; RGCs, retinal ganglion cells. Comparison between groups was statistically significant.

### 3.1. Oxidative stress

**Figure 1:**
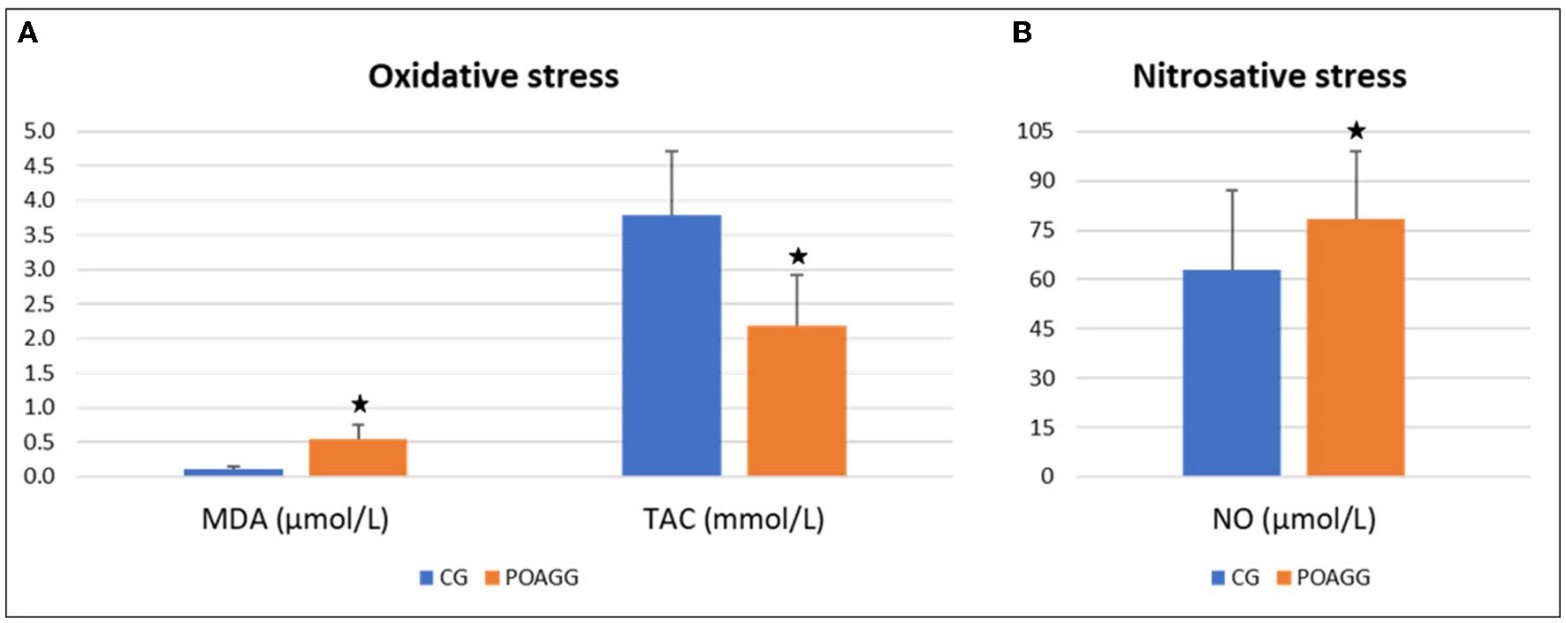
Oxidative stress markers in aqueous humor. POAGG, primary open-angle group; CG, comparative group; MDA, malondialdehyde; TAC, total antioxidant capacity; NO, nitric oxide. The star shows significant differences (*p* < 0.05).

When the antioxidant defense mechanisms fail, all biomolecules (lipids, proteins, nucleic acids, etc.) are susceptible to be attacked by reactive oxygen species (ROS), and probably lipids are the most prone to undergo oxidation, in a process named oxidative stress (OS). Main lipid peroxidation by-products, the total antioxidant status, and the nitrosative stress markers were determined in the study participants' aqueous humor; the results are highlighted in Figure 1.

The MDA levels were significantly higher in the aqueous humor from the POAGG than in the CG (*p* < 0.001). The TAC was considerably lower in the aqueous humor from the POAGG compared to the CG (< 0.001). As a marker of nitrosative stress, the ON levels were significantly higher in the aqueous humor from the glaucoma patients than in the comparatives (*p* < 0.001).

### 3.2. Neuroinflammation

Cytokines are small proteins relevant to cell signaling. Pivotal pro-inflammatory cytokines, including IL-1, IL-6, and TNF-α, exert their effects through type I cytokine receptors (CCR1). These cytokines are essential for coordinating cell-mediated immune response.

**Figure 2:**
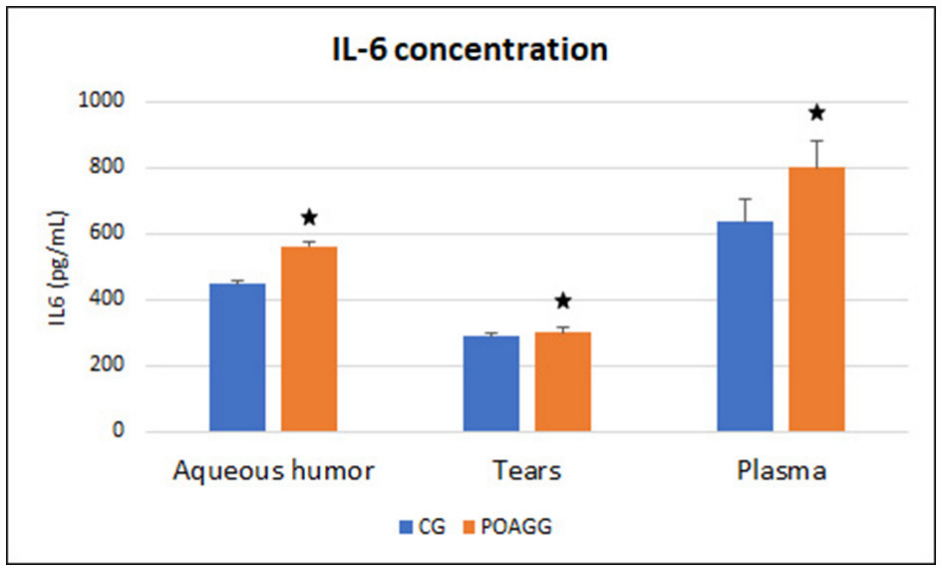
Interleukin-6 concentration in aqueous humor, tear, and plasma samples. POAGG, primary open-angle group; CG, comparative group. The star shows significant differences (*p < * 0.05).

In this study, the cytokine levels were assayed by the IL-6 expression in the tear film, aqueous humor, and plasma samples of our study participants, and the results are shown in Figure 2. Data showed that IL-6 was significantly higher in the tear samples of the glaucoma patients vs. the comparatives (*p* < 0.0001). The IL-6 levels were significantly higher in the aqueous humor of the POAGG vs. the CG (*p* < 0.001). Finally, the plasma IL-6 was significantly higher in the POAGG than in the CG (*p* < 0.001).

### 3.3. Vascular endothelial dysfunction

The vasoconstriction is modulated by the endothelial cells, acting as counterparts of the opposed molecule, the NO, that, in turn, modulates vasodilation. The ET1 is a vasoconstrictor that contributes to the vascular tone and regulates cell proliferation (activating two receptors, ETA, and ETB). ET1 expression levels were significantly higher in the aqueous humor from the POAGG in comparison with the GG individuals operated on cataracts (*p* = 0.001), as compiled in Table 3.

**Table 3:** Biochemical parameters on the vascular endothelium, neurotrophins, and neurotransmitters in the aqueous humor of the participants.

**Parameter**	**POAGG**	**CG**	***p*-value**
ET-1 (ng/ml)	2.75 ± 0.30	1.35 ± 0.16	0.0001
BDNF (pg/ml)	95.75 ± 14.85	111.30 ± 11.43	0.001
5-HT (ng/ml)	2.838 ± 220.61	3.076 ± 98.76	0.820
5-HIAA (ng/ml)	22.87 ± 6.51	19.10 ± 4.66	0.016

POAGG, primary open-angle glaucoma group; CG, comparative–control group; ET-1, endothelin-1; BDNF, brain-derived neurotrophic factor; 5-HT, 5-hydroxy tryptamine/serotonin; 5-HIAA, 5-hydroxyindoleacetic acid. All biochemical parameters were statistically significant, except the 5-HIAA that lacked significance.

### 3.4. Neurotrophins

Neurotrophins or neurotrophic growth factors are essential molecules involved in the development, maintenance, and function of the nervous system. The BDNF is a protein encoded by the BDNF gene with the central role of regulating plastic changes in the adult brain, including regulation of trafficking, phosphorylation, synapsis strength, etc.

The assayed BDNF concentration in the aqueous humor (Table 3) demonstrated significantly lower values of the assayed neurotrophin in the POAGG than in the CG (*p* = 0.001).

### 3.5. Neurotransmitters

Neurotransmitters are chemical messenger molecules released by neurons from the synaptic vesicles into the synapse and from here to the subsequent neurons. Serotonin, an indolamine, is an inhibitory neurotransmitter that regulate mood, anxiety, appetite, pain, sleep patterns, and sexuality. Serotonin is a precursor of melatonin. Melatonin has an inhibitor effect on the levels of nitric oxide.

Regarding the plasma serotonin levels in the study participants, when comparing the POAGG with the CG, 5-HT concentration was noticeably lower in the glaucoma patients (*p* < 0.001). Both the study groups had an inverse correlation between age and plasma 5-HT levels in (Pearson correlation coefficient: −0.191; *p* = 0.028). In addition, when analyzing the 5-HT plasma values with the perimetric evaluation of the right and left eyes (RE/LE) of the POAGG, plasma serotonin levels were significantly lower in the glaucomatous eyes (RE: 125.72 ± 65.46 ng/ml; LE: 128.84 ± 73.91 ng/ml; (*p* < 0.001)].

The aqueous humor concentrations of the assayed neurotransmitter are included in Table 3. 5-HT was noticeably lower in the glaucoma patients than in the comparatives (*p* = 0.820). In addition, 5-HIAA concentration, as a serotonin breakdown product, displayed significantly higher values in the aqueous humor of the POAGG vs. the CG (0.016). The 5-HT turnover (5-HIAA/5-HT) was higher in the GG than in the glaucoma patients (POAGG: 14.050 ng/ml vs. CG: 12.684 ng/ml; *p* = 0.598). In addition, the correlation between 5-HT and 5-HIAA was assessed using Pearson's correlation coefficient, and the levels of 5-HT and 5-HIAA were associated with POAGG: Pearson = −0.756 (*p* = 0.021; as compared to the CG of patients operated of cataracts: Pearson = −0.613 (*p* = 0.028).

### 3.6. Gene expression

**Figure 3:**
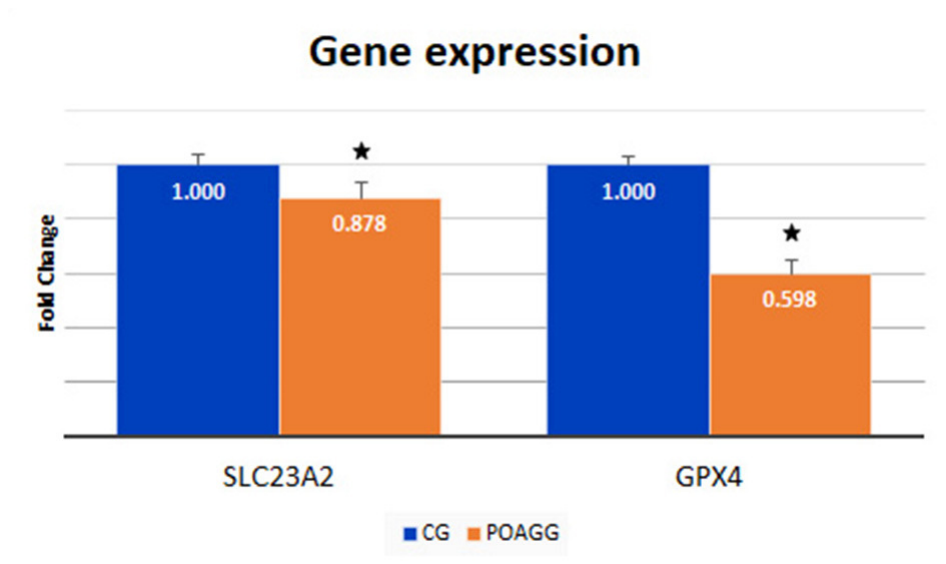
Expression of SLC23A2 and GPX4 genes in blood samples of patients with primary open-angle glaucoma and healthy subjects. POAGG, primary open-angle group; CG, control group. The star shows significant differences (*p* < 0.05).

The role of OS in POAG has been widely investigated. Moreover, many studies have shown the effect of antioxidant supplementation in glaucoma. Goyal et al. (55) demonstrated that vitamin C levels in the aqueous humor of POAG patients were significantly lower than those obtained from comparative individuals operated on cataracts. Similarly, our group found substantially lower levels of vitamin C, GPX, and other antioxidants in the aqueous humor of POAG patients (56). In this study, we assayed the expression levels of two genes involved in OS, the solute carrier family 23—nucleobase transporters—member 2 (SLC23A2) gene, which is the main effector of L-ascorbic acid transmembrane transporter activity, and the glutathione peroxidase 4 (GPX4) gene, also named GSHPx-4, that is implicated in catalyzing the reduction of hydrogen peroxide (H_2_O_2_) by glutathione with the primary purpose of protecting the cells against oxidative attack. We determined the SLC23A2 gene expression in the aqueous humor of the POAG patients, which was significantly lower than that in the CG (*p* < 0.001) (Figure 3). Also, the GPX4 gene expression levels in the aqueous humor of the POAGG were significantly lower than in the CG (*p* < 0.001) (Figure 3).

### 3.7. Micro-RNAs fingerprint

**Figure 4:**
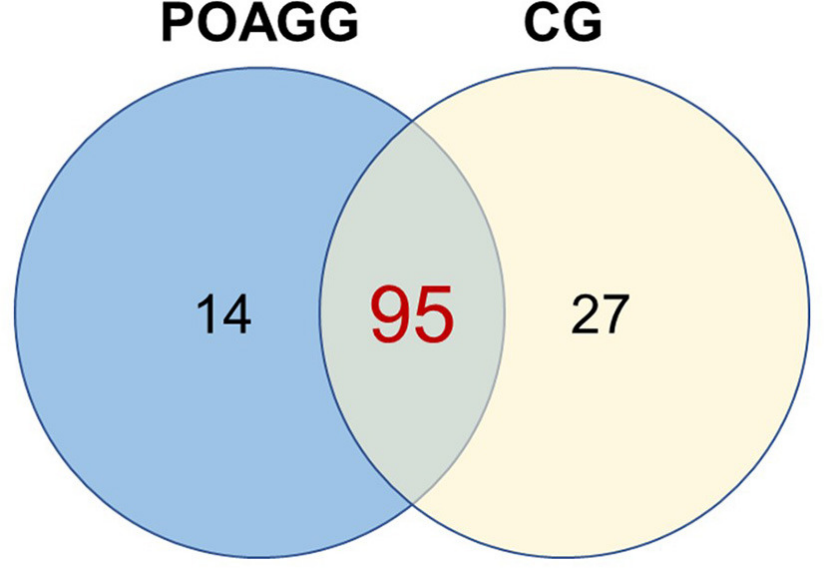
Number of miRNAs identified in tear samples from primary open-angle glaucoma and ocular hypertension patients. POAGG, primary open-angle group; CG, comparative group.

**Figure 5:**
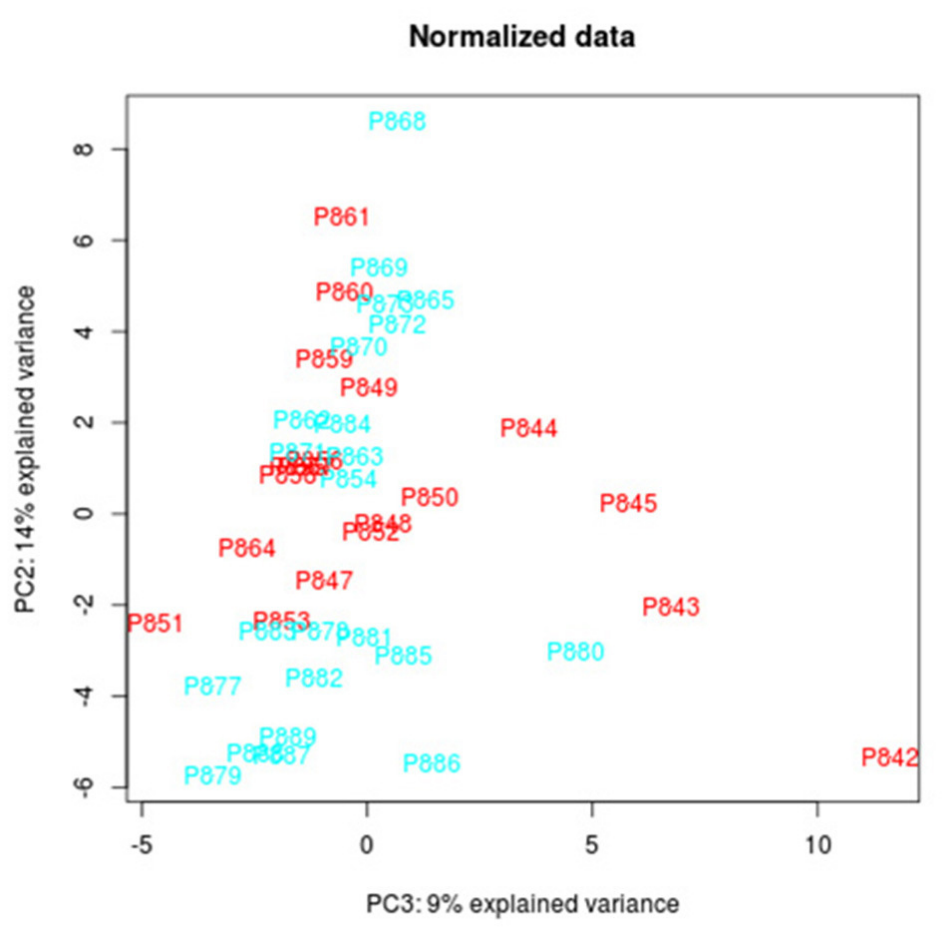
Principal component analysis (PCA) from all the miRNAs identified in the tear samples from the 2 study groups.

**Figure 6:**
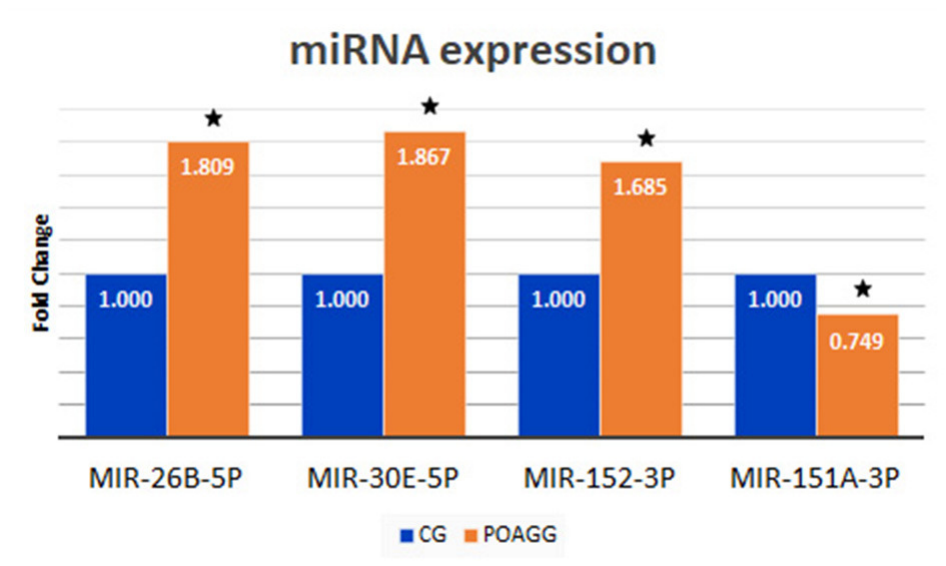
Tear expression of the 4 miRNAs with higher area under the curve from the 2 study groups. POAGG, primary open-angle group; CG, comparative group. The star shows significant differences (*p* < 0.05).

The total RNA extraction in the tear film of the study participants, followed by the construction of libraries, and the analyses by next-generation sequencing (NGS) were carried out, and 122 miRNAs were identified in tears, of which 95 were expressed in both POAG patients and comparative subjects (Figure 4). Bioinformatic analyses permitted to create the PCA of all miRNAs that were identified in tear samples of the participants (Figure 5). Moreover, the more specific PCA (Figure 5) permitted to identify the miRNAs that differentially expressed between groups, and those with the higher area under the curve (Table 4) were hsa-miR-26b-5p (involved in cell proliferation and apoptosis), hsa-miR-152-3p (regulator of cell proliferation, and extracellular matrix expression), hsa-miR-30e-5p (regulates autophagy and apoptosis), and hsa-miR-151a-3p (regulates myoblast proliferation). Figure 6 shows the expression of these miRNAs in tears of POAG patients vs. comparative subjects. In Table 4, miRNAs with the higher statistical power for the differential expression between groups (fold-change between groups) are shown. In Table 5, surrogated biological actions of the four miRNAs identified in the tears of the study participants are reflected.

**Table 4:** miRNAs with the higher statistical power for the differential expression between groups (fold change between groups).

**miRNA ID**	**Fold change^§^(POAGG vs. CG)**	*p*-value^*^	**AUC**
hsa-miR-26b-5p	1.809	0.012	0.81693
hsa-miR-30e-5p	1.867	0.005	0.76201
hsa-miR-151a-3p	0.749	0.009	0.75972
hsa-miR-152-3p	1.685	0.004	0.75743

miRNA, microRNA; ID, identification; POAGG, primary open-angle glaucoma group; CG, comparative group; AUC, area under the curve. ^§^CG was formed by ocular hypertension patients and was set as the reference group. ^*^Significant when *p* = 0.05.

**Table 5:** Biological processes significantly associating primary open-angle glaucoma and miRNAs.

**BP ID**	**BP name**	**N**	*p*-value^*^
GO:0009101	Glycoprotein biosynthetic process	21	0.001
GO:1901137	Carbohydrate derivative biosynthetic process	27	0.002
GO:0030154	Cell differentiation	193	0.006
GO:0007166	Cell surface receptor signaling pathway	138	0.009
GO:0009628	Response to abiotic stimulus	50	0.011
GO:0016477	Cell migration	61	0.018
GO:0048870	Cell motility	63	0.024
GO:1902533	Positive regulation of intracellular signal transduction	41	0.029
GO:1901135	Carbohydrate derivative metabolic process	79	0.03

miRNA, microRNA; BP, biological process; ID, identification; GO, gene ontology.

* Significant when *p* = 0.05.

**Figure 7:**
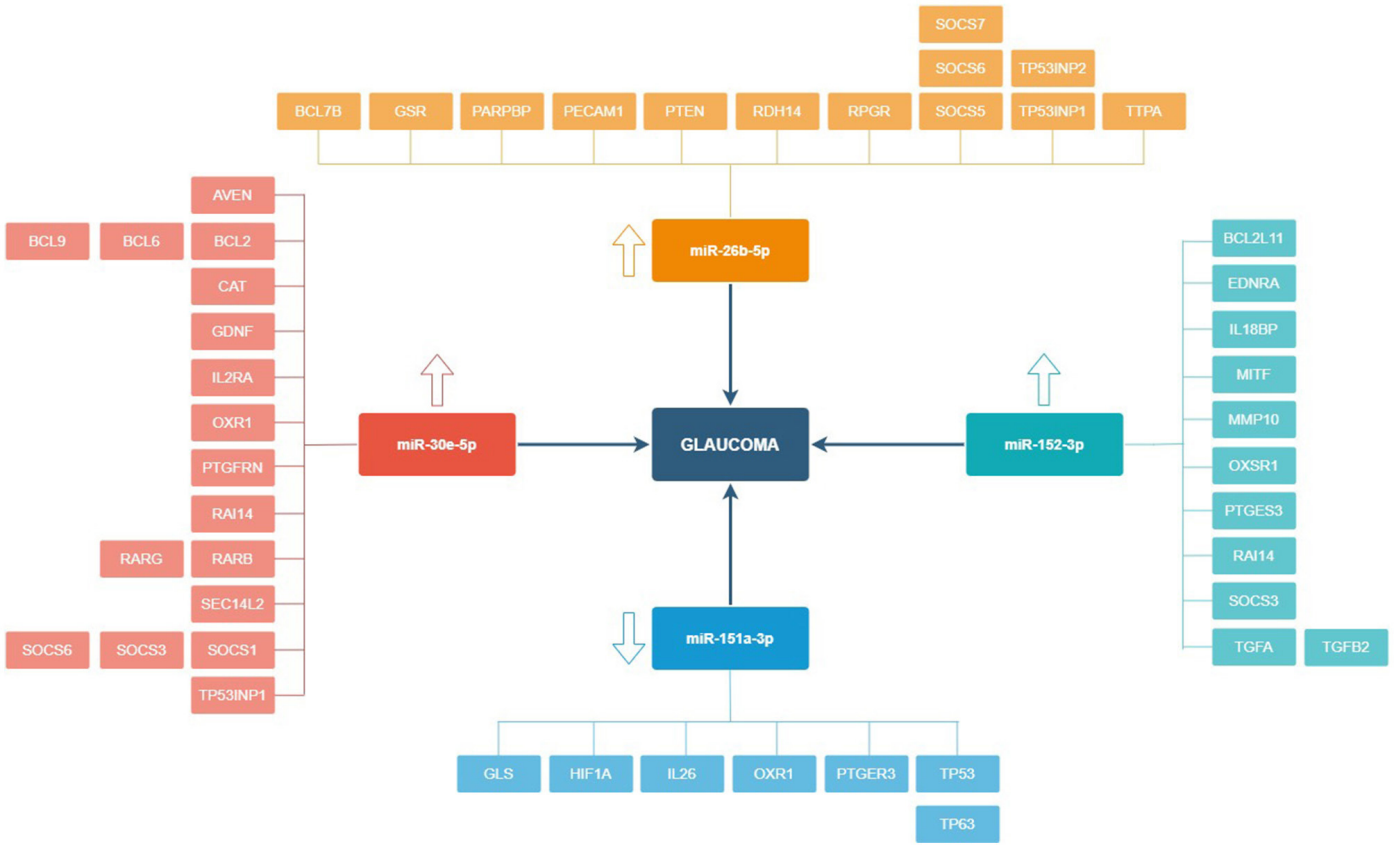
miRNAs and main target genes in relation to primary open-angle glaucoma. The 4 miRNAs included in this figure are those with significantly different expression between groups and with greater area under the curve. Target genes have been obtained from the MicroRNA Target Prediction Database (https://mirdb.org) and TargetScanHuman (https://www.targetscan.org/vert_80).

We designed a new illustration (Figure 7) with the 4 miRNAs with higher area under the curve and their glaucoma-related target genes, identified in the following databases: https://mirdb.org/ and https://www.targetscan.org/.

**Figure 8:**
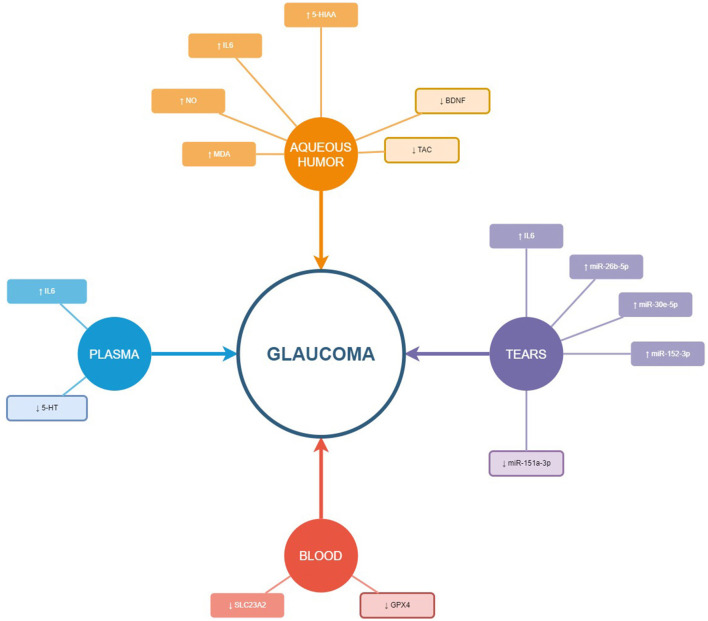
Schematic illustration of the biomarkers studied in the different samples (aqueous humor, tears, plasma, and blood). The up-arrow shows over expression and the down-arrow under expression of the biomarker in glaucoma compared to control or comparative group. The miRNA included in this figure are those 4 with significantly different expression between groups and higher area under the curve.

In summary, we prepared a new figure to reflect the expression trend of the biomarkers obtained from the tears, plasma, blood, and aqueous humor samples of the study participants (Figure 8).

## 4. Discussion

In this study, we explored some classical and emergent biomarkers of POAG in 584 men and women volunteers pertaining to a Spanish ophthalmological population aged 40–80 years, with the primary goal of disclosing the importance of identifying glaucoma biomarkers with the highest sensitivity and specificity to pick out people at risk of glaucoma development and progression, to better fight against glaucoma OND and blindness.

To update the clinical and biochemical-molecular-genetic glaucoma pathogenesis, we collected tears, aqueous humor, and blood samples (whenever possible, one or more than this type of sample per patient) from the POAGG and GC study participants to be processed for identifying POAG biomarkers. For this purpose, we explored several biological pathways: apoptosis, inflammation, neurotransmitter/neurotrophin alteration, oxidative stress, specific gene expression, micro-RNAs fingerprint and its biological targets, and vascular endothelial dysfunction. Thus, we obtained the following biochemical, molecular, and genetic biomarkers that showed a differential expression profile between the groups of participants: MDA, TAC, ON, IL-6, ET1, BDNF, 5-HT, 5-HIAA, the SLC23A2 and GPX4 genes, and the miRNAs hsa-miR-27a-3p, hsa-miR-152-3p, hsa-miR-151a-3p, and hsa-miR-1307-3p.

Scientific evidence supports the role of both oxidative and nitrosative stress in POAG pathogenesis (27–32, 61–63). The following are among the most relevant hypothesis. (1) The trabecular meshwork morphology and function are significantly compromised by ROS. (2) Resistance to aqueous humor outflow in the anterior eye chamber is higher when ROS are present. (3) Main OS parameters are noticeably altered in tears and in aqueous humor of POAG patients. (4) Some specific antioxidant enzymes and vitamins are significantly reduced in the aqueous humor of POAG patients, (5) Oxidative DNA damage significantly correlates with elevated IOP in POAG patients. (6) Specific candidate genes are involved susceptibility to ROS-induced damage in POAG. (7) The anterior and posterior eye segments suffer OS attack in the glaucoma course. In this study, it has been found among the participants that a disbalance between the pro-oxidants and antioxidants in the aqueous humor of the POAG patients with respect to the CG (see Figure 1). In addition, the mean age of the glaucoma participants was 70.03 ± 9.23 years, which is a relevant risk factor for POAG, as well as for OS (27–29, 64). The OS and its downstream effectors have been involved in ocular diseases, such as dry eyes, corneal dystrophies, uveitis, cataracts, diabetic retinopathy, age macular degeneration, retinitis pigmentosa, toxic neuropathies, and others. Despite this, the precise signaling pathways regulated by ROS are, however, not widely known.

Pathogenic glaucoma milestones are the progressive damage and apoptotic death of the RGCs and degeneration of the ONH axons (1, 3, 5, 65). Epidemiological and animal model studies fully demonstrated that neuroinflammation plays a vital role in POAG (21.24). The OS can induce neuroinflammation in the glaucoma course, through a variety of biological pathways, among them: the secretion of pro-inflammatory cytokines from the retinal glia (13, 65). The OS can activate the inflammasome (66–68). Pro-inflammatory cytokines increase phagocytosis in glaucoma eyes (69). In this study, we demonstrated that the IL-6 showed significantly differential expression values in tears, aqueous humor, and plasma from the POAGG concerning the CG.

Endothelial cells intervene in regulating blood flow. Changes in the endothelial cells and the blood vessels are fully involved in the pathogenic mechanisms of a wide variety of diseases: diabetes, stroke, cardiopathies, venous thrombosis, chronic kidney failure, tumor growth, and metastasis (70). Endothelial dysfunction early occurs in the vascular complications associated with the above diseases and has been linked to the decreased bioavailability of specific vasodilators, such as NO. Nevertheless, it has been well established that the relevant role of the enhanced endogenous activity of ET-1, a vasoconstrictor, pro-inflammatory, and mitogenic endothelial peptide in specific human diseases related to endothelial dysfunction (71). Despite the importance of the vascular endothelium and the role of ET-1 and NO, scarce but exciting research has been done in relation to POAG pathogenesis (72, 73).

In this study, we have shown that ET1 and NO levels were significantly higher in the aqueous humor from the POAGG than that in GG, as reflected in Table 3 and Figure 1. It has been described that increased ET-1 availability is rather a consequence of reduced NO levels (74). We venture that an imbalance between ET1/NO can be involved in developing endothelial dysfunction and altered modulation of ocular blood flow. and aqueous humor homeostasis.

A wide variety of molecular signals promote RGC and optic fibers death in glaucoma OND, among them it has to be considered: OS, INF, mitochondrial failure, excitotoxicity, neurotrophin deprivation, axonal transport dysfunction, apoptosis, neuroglia alterations, synaptic loss, etc. (31, 35) Trying to understand the involvement of neurotrophins in POAG could help improve knowledge for better eye and vision care in glaucoma. In this study, we have found that BDNF levels in the aqueous humor (refer to Table 3) were significantly lower in the POAGG than in the CG.

Neurotransmitters are essential for visual function. Although specific neurotransmitters, such as the cholinergic drugs, have been used in hypotensive glaucoma treatment (75), less is known about the role of other neurotransmitters in enhancing neuron survival and vision care in glaucoma. It has been recently reported that Coenzyme Q10 (an electron carrier from complexes I and II to complex III, with an essential role in adenosine triphosphate synthesis, and an important antioxidant) and Citicoline (a nootropic agent con numerous beneficial effects in the CNS), eventually combined, in the prevention of glaucoma OND (76). Herein, we have shown that the expression levels of serotonin in plasma samples and aqueous humor were noticeably lower in the POAGG vs. the CG. Furthermore, when analyzing the inhibitory neurotransmitter plasma levels with the perimetric evaluation of both eyes of the study participants, plasma serotonin concentration was significantly lower in the glaucoma eyes than in the CG. We propose that serotonin deserves further research for potential use as a diagnostic glaucoma biomarker.

Gene expression of some genes related to OS has been studied in this study. Previously, our group and others found significantly lower levels of vitamin C, GPX, and other antioxidants in the aqueous humor of POAG patients (56, 57) demonstrating that vitamin C levels in the aqueous humor of POAG patients were significantly lower than those obtained from comparative individuals operated of cataracts. Majsterek et al. (77) reported significantly lower levels of GPS in the aqueous humor of POAG patients compared with the CG. These results support the critical role of genes related to the OS physiopathology and antioxidant defenses in POAG.

miRNAs are small non-coding RNA molecules that regulate gene expression, and have been involved in health and disease, including glaucoma (78). In this study, we have successfully profiled the tear miRNAs fingerprint from POAG patients with respect to the CG, and 4 specific miRNAs were the strongest candidates to be diagnostic glaucoma biomarkers. Those miRNAs are: hsa-miR-26b-5p (involved in cell proliferation and apoptosis), hsa-miR-152-3p (regulator of cell proliferation, and extracellular matrix expression), hsa-miR-30e-5p (that regulates autophagy and apoptosis), and hsa-miR-151a-3p (regulator of myoblast proliferation). In Figure 6, the differential expression of these miRNAs in tears of POAG patients vs. comparative subjects can be fully appreciated. Also, new biotherapies for POAG can arise from the aforementioned identified miRNAs.

Regarding the biological samples (79) used in this study, we emphasize that tears are painless and relatively easy to collect, store, manipulate, and process, and are instrumental samples to identify biochemical, molecular, and genetic biomarkers, that differentially express themselves in the POAG patients and the comparative-control individuals. In contrast, aqueous humor requires to be collected in surgical conditions. Still, more volume of sample (100 μl) than in the case of the reflex tears (30 μl) can be collected from each eye at the very beginning of surgery, which benefits the number of molecules to be determined. This also happens with the blood samples, which need to be done by the nursing staff who can collect 10–20 ml from each person.

Study limitations are as follows. (1) Volunteers may have underreported their personal and familiar data for the clinical history (either consciously or because of recall bias). (2) Glaucoma medications were recorded but not included in the final statistics. (3) This study produced much information that was statistically processed by generating a large volume of data. Thus, we concentrated on the primary study purposes, and some of the information and data acquired were finally excluded from the final statistical processing. Correspondingly, some actions were carried out in order to reduce the study limitations and to achieve the best results. (1) It was better to revise and confirm the patient data collection. (2) Any discrepancy in the recruitment of participants, data screening, and results obtained was discussed by our team. We know that the study results could have reflected any of the described limitations. Nevertheless, to confirm coherence in the collected information, both the data scrubbing and normalization were independently performed by two researchers. With the aforementioned actions, we warrant the strength of our data power.

## 5. Conclusion

Studies to date have been adding classical biomarkers for POAG. Based on our present study, several potential solid biomarker candidates have emerged in relation to the most relevant POAG pathogenic mechanisms: OS, INF, vascular endothelium, neurotrophins and neurotransmitters, specific genes, and miRNAs. Therefore, the following biochemical, molecular, and genetic biomarkers have been considered for diagnosis, management, and assessment of therapeutic effects of POAG: MDA, TAC, NO, IL-6, ET-1, 5-HT, and 5-HIAA, SLC25A2 gene, GPX4 gene and the miRNAs hsa-miR-26b-5p, hsa-miR-152-3p, hsa-miR-30e-5p, and hsa-miR-151a-3p. Viewing the complexity of the pathogenic mechanisms of POAG, we suggest that the design and development of blended biomarkers is a more appropriate solution in ophthalmological practice for early diagnosis and to predict therapeutic response in glaucoma patients.

## Data availability statement

The data for this article is available in Dryad Digital Repository: Zanon-Moreno, Vicente; Pinazo-Duran, Dolores (2023), miRNAs in primary open-angle glaucoma and ocular hypertension, Dryad, Dataset, https://doi.org/10.5061/dryad.bcc2fqzj1, link: https://datadryad.org/stash/share/Uyjq8RwQTXQ-agNmK1uR1Kl9v6uh9bZ7lR7DjxCq7sU.

## Ethics statement

The studies involving human participants were reviewed and approved by CEIC University Hospital Doctor Peset, Valencia. The patients/participants provided their written informed consent to participate in this study.

## Author contributions

CN and JG-F: study coordinators. All authors contributed to the article and approved the submitted version.

## Glossary

5HIAA, 5-Hydroxy-indoleacetic acid; 5HT, 5-Hydroxy tryptamine/serotonin; AUC, area under the curve; AVEN, apoptosis and caspase activation inhibitor; BCL2, B-Cell CLL/Lymphoma 2; BCL2L11, Bcl-2-Like Protein 11; BCL6, B Cell CLL/Lymphoma 6; BCL7B, B-Cell CLL/Lymphoma 7 Protein Family Member B; BCL9, B-Cell CLL/Lymphoma 9; BCVA, best corrected visual acuity; BDNF, brain-derived neurotrophic factors; CAT, catalase; CCT, central corneal thickness; cDNA, complementary DNA; CG, comparative-control group; CLOCK, circadian locomotor output cycles protein kaput; CYP1B1, cytochrome P450, family 1, subfamily B, polypeptide 1; ECD, electrochemical detection; ECE-1, endothelin converting enzyme 1; EDNRA, endothelin receptor type A; EDTA, ethylene-diamine-tetra-acetic acid; ELISA, enzyme-linked immunosorbent assay; ET1, endothelin 1; GDNF, glial cell-derived neurotrophic factor; GLS, glutaminase; GPX4, glutathione peroxidase 4; GSR, glutathione S-reductase; GSTM1, glutathione S-transferase mu 1; HIF1A, hypoxia inducible factor 1 subunit alpha; HPLC, high-performance liquid chromatography; IL1, interleukin 1; IL17A, interleukin 17A; IL18BP, interleukin 18 binding protein; IL26, interleukin 26; IL2RA, interleukin 2 receptor subunit alpha; IL6, interleukin 6; IL6ST, interleukin 6 cytokine family signal transducer; INF, inflammation; IOP, intraocular pressure; LE, left eye; LogMAR, logarithm of the minimum angle of resolution; MDA, malondialdehyde; MIGS, microincisional glaucoma surgery; miRNA, microribonucleic acid; MITF, microphthalmia-associated transcription factor; MMP10, matrix metallopeptidase 10; MYOC, myocilin; NT3, neurotrophin 3; NT4/5, neurotrophin 4/5; OCT, optical coherence tomography; OCTA, optical coherence tomography angiography; OHT, ocular hypertension; ON, nitric oxide; OND, optic nerve degeneration; OPTN, optineurin; OS, oxidative stress; OXR1, oxidation resistance 1; OXSR1, oxidative stress-responsive kinase 1; PARPBP, PARP1 binding protein; PECAM1, platelet and endothelial cell adhesion molecule 1; POAG, primary open-angle glaucoma; POAGG, primary open-angle glaucoma group; PTEN, phosphatase and tensin homolog; PTGER3, prostaglandin E receptor 3; PTGFRN, prostaglandin F2 receptor inhibitor; PTGS2, prostaglandin-endoperoxide synthase 2; RAI14, retinoic acid-induced 14; RARB, retinoic acid receptor beta; RARG, retinoic acid receptor gamma; RARRES1, retinoic acid receptor responder 1; RDH14, retinol dehydrogenase 14; RE, right eye; RGC, retinal ganglion cells; RIN, RNA integrity number; RNFL, retinal nerve fiber layer; ROC, receiver operating characteristic; RPGR, retinitis pigmentosa GTPase regulator; SD, standard deviation; SEC14L2, SEC14-like lipid binding 2 (tocopherol-associated protein 1); SLC23A2, solute carrier family 23 member 2; SOCS1, suppressor of cytokine signaling 1; SOCS3, suppressor of cytokine signaling 3; SOCS5, suppressor of cytokine signaling 5; SOCS6, suppressor of cytokine signaling 6; SOCS7, suppressor of cytokine signaling 7; TAC, total antioxidant capacity; TBA, thiobarbituric acid; TBARS, thiobarbituric acid reactive substances; TGFA, transforming growth factor alpha; TGFB2, transforming growth factor beta 2; TNF-α, tumor necrosis factor alpha; TP53, tumor protein P53; TP53INP1, tumor protein P53; inducible nuclear protein 1; TP53INP2, tumor protein P53 inducible nuclear protein 2; TP63, tumor protein P63; TTPA, alpha tocopherol transfer protein; VD, vascular dysfunction; WDR36, WD repeat Domain 36.

## References

[1] European Glaucoma Society. Terminology guidelines for glaucoma. 5th ed. (2020). Available online at: https://www.eugs.org/eng/egs_guidelines_reg.asp (accessed February 01, 2023).

[2] Wu Z Medeiros FA. Recent developments in visual field testing for glaucoma. Curr Opin Ophthalmol. (2018) 29:141–6. 10.1097/ICU.0000000000000461 29256895

[3] Brusini P. Global Glaucoma Staging System (GGSS): A New Method to Simultaneously Assess the Severity of Both Functional and Structural Damage in Glaucoma. J Clin Med. (2021) 10:4414. 10.3390/jcm10194414 34640431 PMC8509816

[4] Gallo Afflitto G Aiello F Cesareo M Nucci C. Primary Open Angle Glaucoma Prevalence in Europe: A Systematic Review and Meta-Analysis. J Glaucoma. (2022) 31:783–8. 10.1097/IJG.0000000000002083 35980843

[5] Jackson AB Martin KR Coote MA Medeiros FA Girkin CA Fazio MA., Fast progressors in glaucoma: prevalence based on global and central visual field loss. Ophthalmology. (2023) 63:1252–A0392. 10.1016/j.ophtha.2023.01.008 36693593 PMC10121866

[6] Pinazo-Duran MD Zanón-Moreno V García-Medina JJ Arévalo JF Gallego-Pinazo R Nucci C. Eclectic ocular comorbidities and systemic diseases with eye involvement: a review. Biomed Res Int. (2016) 6215745:1–10. 10.1155/2016/6215745 27051666 PMC4808667

[7] Cesareo M Ciuffoletti E Ricci F Missiroli F Giuliano MA Mancino R. Visual disability, and quality of life in glaucoma patients. Prog Brain Res. (2015) 221:359–74. 10.1016/bs.pbr.2015.07.003 26518087

[8] Jin J. Screening for Primary Open-Angle Glaucoma. JAMA. (2022) 327:2030. 10.1001/jama.2022.7531 35608579

[9] Rivera JL Bell NP Feldman RM. Risk factors for primary open angle glaucoma progression: what we know and what we need to know. Curr Opin Ophthalmol. (2008) 19:102–6. 10.1097/ICU.0b013e3282f493b3 18301282

[10] Lee SS Mackey DA. Glaucoma - risk factors and current challenges in the diagnosis of a leading cause of visual impairment. Maturitas. (2022) 163:15–22. 10.1016/j.maturitas.2022.05.002 35597227

[11] Garcia-Villanueva C Milla E Bolarin JM García-Medina JJ Cruz-Espinosa J Benítez-del- Castillo J. Impact of systemic comorbidities on ocular hypertension and Open-Angle glaucoma, in a population from Spain and Portugal. J Clin Med. (2022) 11:5649. 10.3390/jcm11195649 36233515 PMC9570920

[12] Cordeiro MF Guo L Luong V Harding G Wang W Jones HE. Real-time imaging of single nerve cell apoptosis in retinal neurodegeneration. Proc Natl Acad Sci U S A. (2004) 101:13352–6. 10.1073/pnas.0405479101 15340151 PMC516570

[13] Pinazo-Durán MD Muñoz-Negrete FJ Sanz-González SM Benítez-Del-Castillo J Giménez- Gómez R Valero-Velló M. The role of neuroinflammation in the pathogenesis of glaucoma neurodegeneration. Prog Brain Res. (2020) 256:99–124. 10.1016/bs.pbr.2020.07.004 32958217

[14] Gonzalez-Hernandez M Gonzalez-Hernandez D Perez-Barbudo D Rodriguez-Esteve P Betancor-Caro N Gonzalez de la Rosa M. Fully Automated Colorimetric Analysis of the Optic Nerve Aided by Deep Learning and Its Association with Perimetry and OCT for the Study of Glaucoma. J Clin Med. (2021) 10:3231. 10.3390/jcm10153231 34362014 PMC8347493

[15] Gordon MO Beiser JA Brandt JD Heuer DK Higginbotham EJ Johnson CA et al. The Ocular Hypertension Treatment Study: Baseline factors that predict the onset of primary open-angle glaucoma. Arch Ophthalmol. (2002) 120:714–20. 10.1001/archopht.120.6.714 12049575

[16] Kass MA Heuer DK Higginbotham EJ Johnson CA Keltner JL Miller JP et al. The Ocular Hypertension Treatment Study: A randomized trial determines that topical ocular hypotensive medication delays or prevents the onset of primary open-angle glaucoma. Arch Ophthalmol. (2002) 120:701–13. 10.1001/archopht.120.6.701 12049574

[17] Weinreb RN Aung T Medeiros FA. The pathophysiology and treatment of glaucoma: A review. JAMA. (2014) 311:1901–11. 10.1001/jama.2014.3192 24825645 PMC4523637

[18] Storgaard L Tran TL Freiberg JC Hauser AS Kolko M. Glaucoma clinical research: trends in treatment strategies and drug development. Front Med. (2021) 8:733080. 10.3389/fmed.2021.733080 34589504 PMC8473801

[19] Cardigos J Ferreira Q Crisóstomo S Moura-Coelho N Cunha JP Pinto LA. Nanotechnology-ocular devices for glaucoma treatment: a literature review. Curr Eye Res. (2019) 44:111–7. 10.1080/02713683.2018.1536218 30309248

[20] Erb C. New therapeutic concepts in glaucoma treatment. Ophthalmologe. (2021) 118:429–30. 10.1007/s00347-021-01368-7 33961113

[21] Vecino E. Sharma S.C. InTech; Glaucoma animal models. In: Glaucoma-basic clinical concepts. (2011). Available online at: https://www.intechopen.com/download/pdf/23828

[22] Mayordomo-Febrer A López-Murcia M Morales-Tatay JM Monleón-Salvado D Pinazo-Durán MD. Metabolomics of the aqueous humor in the rat glaucoma model induced by a series of intracamerular sodium hyaluronate injection. Exp Eye Res. (2015) 131:84–92. 10.1016/j.exer.2014.11.012 25479046

[23] Vidal-Sanz M Valiente-Soriano FJ Ortín-Martínez A Nadal-Nicolás FM Jiménez-López M Salinas-Navarro M. Retinal neurodegeneration in experimental glaucoma. Prog Brain Res. (2015) 220:1–35. 10.1016/bs.pbr.2015.04.008 26497783

[24] Evangelho K Mastronardi CA de-la-Torre A. Experimental models of glaucoma: a powerful translational tool for the future development of new therapies for glaucoma in humans-a review of the literature. Medicina (Kaunas). (2019) 55:280. 10.3390/medicina55060280 31212881 PMC6630440

[25] Fernandez-Albarral JA Ramírez AI de Hoz R Salazar JJ. Retinal microglial activation in glaucoma: evolution over time in a unilateral ocular hypertension model. Neural Regen Res. (2022) 17:797–9. 10.4103/1673-5374.322454 34472476 PMC8530147

[26] Noailles A Kutsyr O Mayordomo-Febrer A Lax P López-Murcia M Sanz-González SM. Sodium Hyaluronate-Induced Ocular Hypertension in Rats Damages the Direction-Selective Circuit and Inner/Outer Retinal Plexiform Layers. Invest Ophthalmol Vis Sci. (2022) 63:2. 10.1167/iovs.63.5.2 35503230 PMC9078050

[27] Zanon-Moreno V Marco-Ventura P Lleo-Perez A Pons-Vazquez S Garcia-Medina JJ Vinuesa-Silva I. Oxidative stress in primary open-angle glaucoma. J Glaucoma. (2008) 17:263–84. 10.1097/IJG.0b013e31815c3a7f 18552610

[28] Pinazo-Durán MD Zanón-Moreno V Gallego-Pinazo R García-Medina JJ. Oxidative stress and mitochondrial failure in the pathogenesis of glaucoma neurodegeneration. Prog Brain Res. (2015) 220:127–53. 10.1016/bs.pbr.2015.06.001 26497788

[29] Pinazo-Durán MD Shoaie-Nia K Zanon-Moreno V Sanz-Gonzalez SM Del Castillo JB Garcia-Medina JJ. Strategies to reduce oxidative stress in glaucoma patients. Curr Neuropharmacol. (2018) 16:903–18. 10.2174/1570159X15666170705101910 28677495 PMC6120109

[30] Wang Y Huang C Zhang H Wu R. Autophagy in glaucoma: Crosstalk with apoptosis and its implications. Brain Res Bull. (2015) 117:1–9. 10.1016/j.brainresbull.2015.06.001 26073842

[31] Almasieh M Wilson AM Morquette B Cueva Vargas JL Di Polo A. The molecular basis of retinal ganglion cell death in glaucoma. Prog Retin Eye Res. (2012) 31:152–81. 10.1016/j.preteyeres.2011.11.002 22155051

[32] Baudouin C Kolko M Melik-Parsadaniantz S Messmer EM. Inflammation in Glaucoma: From the back to the front of the eye, and beyond. Prog Ret Eye Res. (2021) 83:100916 10.1016/j.preteyeres.2020.100916 33075485

[33] Pasquale LR. Vascular and autonomic dysregulation in primary open-angle glaucoma. Curr Opin Ophthalmol. (2016) 27:94–101. 10.1097/ICU.0000000000000245 26720776 PMC4740225

[34] Alarcon-Martinez L Shiga Y Villafranca-Baughman D Belforte N Quintero H Dotigny F. Pericyte dysfunction and loss of interpericyte tunneling nanotubes promote neurovascular deficits in glaucoma. Proc Natl Acad Sci U S A. (2022) 119:e2110329119. 10.1073/pnas.2110329119 35135877 PMC8851476

[35] Chitranshi N Dheer Y Abbasi M You Y Graham SL Gupta V. Glaucoma Pathogenesis and Neurotrophins: Focus on the Molecular and Genetic Basis for Therapeutic Prospects. Curr Neuropharmacol. (2018) 16:1018–1035. 10.2174/1570159X16666180419121247 29676228 PMC6120108

[36] Taylor S Srinivasan B Wordinger RJ Roque RS. Glutamate stimulates neurotrophin expression in cultured Müller cells. Brain Res Mol Brain Res. (2003) 111:189–97. 10.1016/S0169-328X(03)00030-5 12654519

[37] González-Iglesias H Álvarez L García M Escribano J Rodríguez-Calvo PP Fernández-Vega L. Comparative proteomic study in serum of patients with primary open-angle glaucoma and pseudoexfoliation glaucoma. J Proteomics. (2014) 98:65–78. 10.1016/j.jprot.2013.12.006 24355480

[38] Kumar S Ahmad Malik M Goswami S Sijota R Kaur J. Candidate genes involved in the susceptibility of primary open angle glaucoma. Gene. (2016) 577:119–31. 10.1016/j.gene.2015.11.032 26621382

[39] Shin S Jung Y Uhm H Song M Son S Goo J. Quantification of purified endogenous miRNAs with high sensitivity and specificity. Nat Commun. (2020) 11:6033. 10.1038/s41467-020-19865-9 33247115 PMC7699633

[40] Li X Zhao F Xin M Li G Luna C Li G. Regulation of intraocular pressure by microRNA cluster miR-143/145. Sci Rep. (2017) 7:915. 10.1038/s41598-017-01003-z 28424493 PMC5430458

[41] Peng H Sun YB Hao JL Lu CW Bi MC Song E. Neuroprotective effects of overexpressed microRNA-200a on activation of glaucoma-related retinal glial cells and apoptosis of ganglion cells via downregulating FGF7-mediated MAPK signaling pathway. Cell Signal. (2019) 54:179–90. 10.1016/j.cellsig.2018.11.006 30439502

[42] Benitez-Del-Castillo J Cantu-Dibildox J Sanz-González SM Zanón-Moreno V Pinazo-Duran MD. Cytokine expression in tears of patients with glaucoma or dry eye disease: A prospective, observational cohort study. Eur J Ophthalmol. (2018) 29:437–43. 10.1177/1120672118795399 30175615

[43] Benítez Del Castillo JM Pinazo-Duran MD Sanz-González S Muñoz-Hernández AM Garcia-Medina JJ Zanón-Moreno V. Tear 1H NMR-based metabolomics application to the molecular diagnosis of aqueous tear deficiency and Meibomian gland dysfunction. Ophthalmic Res. (2020) 64:297–309. 10.1159/000510211 32674101

[44] Zhou L Beuerman RW. Tear analysis in ocular surface diseases. Prog Retin Eye Res. (2012) 31:527–50. 10.1016/j.preteyeres.2012.06.002 22732126

[45] Pinazo-Durán MD Zanón-Moreno V García-Medina JJ Gallego-Pinazo R. Evaluation of presumptive biomarkers of oxidative stress, immune response, and apoptosis in primary open-angle glaucoma. Curr Opin Pharmacol. (2013) 13:98–107. 10.1016/j.coph.2012.10.007 23142105

[46] Kambayashi Y Binh NT Asakura HW Hibino Y Hitomi Y Nakamura H. Efficient assay for total antioxidant capacity in human plasma using a 96-well microplate. J Clin Biochem Nutr. (2009) 44:46–51. 10.3164/jcbn.08-162 19177187 PMC2613498

[47] Mas-Bargues C Escrivá C Dromant M Borrás C Viña J. Lipid peroxidation as measured by chromatographic determination of malondialdehyde. Human plasma reference values in health and disease. Arch Biochem Biophys. (2021) 709:108941. 10.1016/j.abb.2021.108941 34097903

[48] Nucci C Di Pierro D Varesi C Ciuffoletti E Russo R Gentile R. Increased malondialdehyde concentration and reduced total antioxidant capacity in aqueous humor and blood samples from patients with glaucoma. Mol Vis. (2013) 19:1841–6. 23946639 PMC3742119

[49] Pinazo-Durán MD García-Medina JJ Bolarín JM Sanz-González SM Valero-Vello M Abellán-Abenza J. Computational analysis of clinical and molecular markers and new theranostic possibilities in primary open-angle glaucoma. J Clin Med. (2020) 9:3032. 10.3390/jcm9093032 32967086 PMC7564865

[50] Ersan S Cigdem B Bakir D Dogan HO. Determination of levels of oxidative stress and nitrosative stress in patients with epilepsy. Epilepsy Res. (2020) 164:106352. 10.1016/j.eplepsyres.2020.106352 32446164

[51] Audhya T Adams JB Johansen L. Correlation of serotonin levels in CSF, platelets, plasma, and urine. Biochim Biophys Acta. (2012) 1820:1496–501. 10.1016/j.bbagen.2012.05.012 22664303

[52] Zanon-Moreno V Garcia-Medina JJ Zanon-Viguer V Moreno-Nadal MA Pinazo-Duran MD. Smoking, an additional risk factor in elder women with primary open-angle glaucoma. Mol Vis. (2009) 15:2953–9. 20057902 PMC2802290

[53] Ulhaq ZS Soraya GV. Aqueous humor interleukin-6 levels in primary open-angle glaucoma (POAG): A systematic review and meta-analysis. Arch Soc Esp Oftalmol (Engl Ed). (2020) 95:315–321. 10.1016/j.oftale.2020.03.003 32414512

[54] Choritz L Mahmoodi B Thieme H. Influence of endothelin-1 in aqueous humor on intermediate-term trabeculectomy outcomes. J Ophthalmol. (2016) 2016:2401976. 10.1155/2016/2401976 26904271 PMC4745626

[55] Igarashi T Nakamoto K Kobayashi M Suzuki H Arima T Tobita Y et al. Brain-derived neurotrophic factor in the aqueous humor of glaucoma patients. J Nippon Med Sch. (2021) 88:128–32. 10.1272/jnms.JNMS.2021_88-305 33980757

[56] Goyal A Srivastava A Sihota R Kaur J. Evaluation of oxidative stress markers in aqueous humor of primary open angle glaucoma and primary angle closure glaucoma patients. Curr Eye Res. (2014) 39:823–9. 10.3109/02713683.2011.556299 24912005

[57] Zanon-Moreno V Asensio-Marquez EM Ciancotti-Oliver L Garcia-Medina JJ Sanz P Ortega-Azorin C. Effects of polymorphisms in vitamin E-, vitamin C-, and glutathione peroxidase-related genes on serum biomarkers and associations with glaucoma. Mol Vis. (2013) 19:231–42. 23401652 PMC3566896

[58] Burgos KL Javaherian A Bomprezzi R Ghaffari L Rhodes S Courtright A. Identification of extracellular miRNA in human cerebrospinal fluid by next-generation sequencing. RNA. (2013) 19:712–22. 10.1261/rna.036863.112 23525801 PMC3677285

[59] Tamkovich S Grigor'eva A Eremina A Tupikin A Kabilov M Chernykh V. What information can be obtained from the tears of a patient with primary open angle glaucoma? Clin Chim Acta. (2019) 495:529–37. 10.1016/j.cca.2019.05.028 31153869

[60] Raga-Cervera J Bolarin JM Millan JM Garcia-Medina JJ Pedrola L Abellán-Abenza J. miRNAs and Genes Involved in the Interplay between Ocular Hypertension and Primary Open-Angle Glaucoma. Oxidative Stress, Inflammation, and Apoptosis Networks. J Clin Med. (2021) 10:2227. 10.3390/jcm10112227 34063878 PMC8196557

[61] Izzotti A Di Marco B De Flora S Saccà S. Open angle glaucoma: epidemiology, pathogenesis and prevention. Recenti Prog Med. (2006) 97:37–45. 16535929

[62] Saccà SC Izzotti A. Oxidative stress and glaucoma: injury in the anterior segment of the eye. Prog Brain Res. (2008) 173:385–407. 10.1016/S0079-6123(08)01127-8 18929123

[63] McMonnies C. Reactive oxygen species, oxidative stress, glaucoma and hyperbaric oxygen therapy. J Optom. (2018) 11:3–9. 10.1016/j.optom.2017.06.002 28760643 PMC5777925

[64] Pinazo-Durán MD Gallego-Pinazo R García-Medina JJ Zanón-Moreno V Nucci C Dolz-Marco R. Oxidative stress and its downstream signaling in aging eyes. Clin Interv Aging. (2014) 9:637–52. 10.2147/CIA.S52662 24748782 PMC3990383

[65] Tezel G Yang X Luo C Peng Y Sun SL Sun D. Mechanisms of immune system activation in glaucoma: oxidative stress-stimulated antigen presentation by the retina and optic nerve head glia. Invest Ophthalmol Vis Sci. (2007) 48:705–14. 10.1167/iovs.06-0810 17251469 PMC2494942

[66] Chi W Li F Chen H Wang Y Zhu Y Yang X. Caspase-8 promotes NLRP1/NLRP3 inflammasome activation and IL-1β production in acute glaucoma. Proc Natl Acad Sci U S A. (2014) 111:11181–6. 10.1073/pnas.1402819111 25024200 PMC4121847

[67] Yerramothu P Vijay AK Willcox MDP. Inflammasomes, the eye and anti-inflammasome therapy. Eye (Lond). (2018) 32:491–505. 10.1038/eye.2017.241 29171506 PMC5848281

[68] Zanon-Moreno V Raga-Cervera J García-Medina JJ Benitez-Del-Castillo J Vinuesa-Silva I Torregrosa S. New horizons for the treatment of glaucoma. I: Neuroinflammation and inflammasomes. Arch Soc Esp Oftalmol (Engl Ed). (2018) 93:e7–9. 10.1016/j.oftale.2018.01.001 29289427

[69] Ahmad A Ahsan H. Biomarkers of inflammation and oxidative stress in ophthalmic disorders. J Immunoassay Immunochem. (2020) 41:257–71. 10.1080/15321819.2020.1726774 32046582

[70] Nishiyama SK Zhao J Wray W Richardson RS. Vascular function and endothelin-1: tipping the balance between vasodilation and vasoconstriction. J Appl Physiol. (2017) 122:354–60. 10.1152/japplphysiol.00772.2016 27909229 PMC5338602

[71] Genovesi S Giussani M Orlando A Lieti G Viazzi F Parati G. Relationship between endothelin and nitric oxide pathways in the onset and maintenance of hypertension in children and adolescents. Pediatr Nephrol. (2022) 37:537–45. 10.1007/s00467-021-05144-2 34085102 PMC8921137

[72] Ghanem AA Elewa AM Arafa LF. Endothelin-1 and nitric oxide levels in patients with glaucoma. Ophthalmic Res. (2011) 46:98–102. 10.1159/000323584 21282966

[73] Dismuke WM Liang J Overby DR Stamer WD. Concentration-related effects of nitric oxide and endothelin-1 on human trabecular meshwork cell contractility. Exp Eye Res. (2014) 120:28–35. 10.1016/j.exer.2013.12.012 24374036 PMC3943640

[74] Bourque SL Davidge ST Adams MA. The interaction between endothelin-1 and nitric oxide in the vasculature: new perspectives. Am J Physiol Reg Integ Comp Physiol. (2011) 300:R1288–95. 10.1152/ajpregu.00397.2010 21368267

[75] Faiq MA Wollstein G Schuman JS Chan KC. Cholinergic nervous system and glaucoma: From basic science to clinical applications. Prog Retin Eye Res. (2019) 72:100767. 10.1016/j.preteyeres.2019.06.003 31242454 PMC6739176

[76] Martucci A Mancino R Cesareo M Pinazo-Duran MD Nucci C. Combined use of coenzyme Q10 and citicoline: A new possibility for patients with glaucoma. Front Med. (2022) 9:1020993. 10.3389/fmed.2022.1020993 36590976 PMC9797721

[77] Majsterek I Malinowska K Stanczyk M Kowalski M Blaszczyk J Kurowska AK. Evaluation of oxidative stress markers in pathogenesis of primary open-angle glaucoma. Exp Mol Pathol. (2011) 90:231–7. 10.1016/j.yexmp.2011.01.001 21241689

[78] Bawazeer A Prince DC. Detection of miRNAs. Methods Mol Biol. (2023) 2630:1–11. 10.1007/978-1-0716-2982-6_1 36689172

[79] Buś MM Allen M. Collecting and preserving biological samples from challenging environments for DNA analysis. Biopreserv Biobank. (2014) 12:17–22. 10.1089/bio.2013.0060 24620766

