# Correction: Biochemical–molecular–genetic biomarkers in the tear film, aqueous humor, and blood of primary open-angle glaucoma patients

**DOI:** 10.3389/fmed.2025.1675008

**Published:** 2025-08-18

**Authors:** Maria D. Pinazo-Durán, Vicente Zanón-Moreno, Carolina García-Villanueva, Alessio Martucci, Cristina Peris-Martínez, Jorge Vila-Arteaga, Jose J. García-Medina, Irene Andrés-Blasco, Alex Gallego-Martínez, Carlo Nucci, Julian García-Feijoo

**Affiliations:** ^1^Ophthalmic Research Unit “Santiago Grisolia” Foundation for Research in Health and Biomedicine, (FISABIO), Valencia, Spain; ^2^Cellular and Molecular Ophthalmobiology Group, Surgery Department, Faculty of Medicine and Odontology, University of Valencia, Valencia, Spain; ^3^Spanish Network of Inflammatory Diseases: REI-RICORS (RD21/0002/0032) of the Institute of Health Carlos III (ISCIII), Spanish Government, Madrid, Spain; ^4^Biosanitary Research Institute, Valencian International University (VIU), Valencia, Spain; ^5^Department of Ophthalmology, The University General Hospital, Valencia, Spain; ^6^Ophthalmology Unit, Department of Experimental Medicine, University of Rome “Tor Vergata”, Rome, Italy; ^7^Medical Ophthalmology FISABIO-FOM Center, Valencia, Spain; ^8^Department of Ophthalmology, University and Polytechnic Hospital “La Fe”, Valencia, Spain; ^9^Department of Ophthalmology, The General University Hospital “Morales Meseguer”, Murcia, Spain; ^10^Department of Ophthalmology and Optometry, University of Murcia, Murcia, Spain; ^11^Department of Ophthalmology, The University Clinic Hospital “San Carlos”, Madrid, Spain

**Keywords:** primary open-angle glaucoma, glaucoma neurodegeneration, biomarkers, molecules, genes, miRNAs

An incorrect Funding statement was provided. The correct funding statement reads: The author(s) declare that financial support was received for the research and/or publication of this article. RICORS-REI (RD21/0002/0032) funded by Instituto de Salud Carlos III (ISCIII) and Next Generation EU funds, which finance the actions of the Recovery and Resilience Mechanism (RRM) have founded part of this study through the financial support given to the research activities of the members pertaining to this Network.

The original version of this article has been updated.

